# Morbid obesity in Taiwan: Prevalence, trends, associated social demographics, and lifestyle factors

**DOI:** 10.1371/journal.pone.0169577

**Published:** 2017-02-02

**Authors:** Heng-Cheng Chang, Hsin-Chou Yang, Hsing-Yi Chang, Chih-Jung Yeh, Hsin-Hung Chen, Kuo-Chin Huang, Wen-Harn Pan

**Affiliations:** 1 Graduate Institute of Life Sciences, National Defense Medical Center, Taipei, Taiwan; 2 Institute of Biomedical Sciences, Academia Sinica, Taipei, Taiwan; 3 Institute of Statistical Science, Academia Sinica, Taipei, Taiwan; 4 Institute of Population Health Sciences, National Health Research Institutes, Miaoli County, Taiwan; 5 School of Public Health, Chung Shan Medical University, Taichung, Taiwan; 6 Department of Nutrition and Health Science, Chang Jung Christian University, Tainan, Taiwan; 7 Department of Family Medicine, National Taiwan University Hospital and College of Medicine, Taipei, Taiwan; Taipei City Hospital, TAIWAN

## Abstract

**Objective:**

Obesity is one of the most important public health issues worldwide. Moreover, an extreme phenotype, morbid obesity (MO) has insidiously become a global problem. Therefore, we aimed to document the prevalence trend and to unveil the epidemiological characteristics of MO in Taiwan.

**Methods:**

Nationally representative samples aged 19 years and above from three consecutive waves of Nutrition and Health survey in Taiwan: 1993–1996, 2005–2008, and 2013–2014 (n = 3,071; 1,673; and 1,440; respectively) were analyzed for prevalence trend. And 39 MO (BMI ≥35 kg/m^2^) cases from the two recent surveys compared with 156 age, gender, and survey-matched normal weight controls (BMI: 18.5–24 kg/m^2^) for epidemiological characteristics study. The reduced rank regression analysis was used to find dietary pattern associated with MO.

**Results:**

The prevalence of overweight and obesity together (BMI ≥24 kg/m^2^) was stabilized in the recent two surveys, but that of MO (0.4%, 0.6%, to 1.4%) and obesity (BMI ≥27 kg/m^2^) (11.8%, 17.9%, to 22.0%) increased sharply. MO cases tended to have lower levels of education, personal income, and physical activity. Furthermore, their dietary pattern featured with a higher consumption frequency of red meat, processed animal products, and sweets/sweetened beverage, but lower frequencies of fresh fruits, nuts, breakfast cereal, and dairy products.

**Conclusion:**

This study documents a polarization phenomenon with smaller proportion of overweight people at the center and higher proportions of normal weight and obesity subjects at two extremes. MO was associated with low socioeconomic status and poor dietary pattern. The obesogenic dietary pattern became more prevalent in later time.

## Introduction

Obesity is a major public health issue in the world. It has been estimated that there are approximately 1.9 billion adults who are either overweight or obese (body mass index, BMI ≥25 kg/m^2^). Among them, over 600 million are obese (BMI ≥30 kg/m^2^)[1]. According to World Health Organization (WHO), this problem of energy imbalance may have contributed to an estimated 3.4 million death each year including those resulted from cardiovascular disease, type 2 diabetes mellitus, and cancers[2, 3]. Quality of life of the obese may also be compromised by conditions such as osteoarthritis, work disability, depression and sleep apnea [2, 3].

The current worldwide prevalence of obesity in adults has been more than doubled in the world since 1980[1].At the same time, the trend of obesity rates seems to be levelling off in some developed countries since 2006[4].On the other hand, the prevalence of an extreme phenotype, morbid obesity (MO) (BMI ≥40 kg/m^2^), is not only persistently rising, but also expected to increase in an accelerating speed in the coming decades [5, 6].Compared to those whom were overweight and obesity, MO population suffer from even a shorter life expectancy, greater severity of many comorbidity, and higher all-cause mortality rate[7, 8],so that associated medical cost and social economic burden are tremendous[9, 10]. And yet weight control measures are less efficient for MO except an extreme measure, bariatric surgery[11].

Taking a preventive standpoint, it is important to understand the trends and risk factors of MO, in order to plan ahead for screening high risk youth, promoting healthy life style, and building supportive environments. However, there are viewpoints such that severe obesity is mainly genetic in origin, not due to the lifestyle and environmental factors[12]. Therefore, in this study we aimed to take advantage of the data from Nutrition and Health Surveys in Taiwan (NAHSIT) to document the MO prevalence trend from near zero to its abrupt appearance and to assess the epidemiological characteristics of MO in Taiwan, an Asian region in a rapid transition.

## Materials and methods

### Study designs and subjects

In this study, use was made of 3 waves of Nutrition and Health Survey in Taiwan (NAHSIT): 1993–1996, 2005–2008, and 2013–2014. The NAHSIT survey has been described elsewhere [13, 14]. In brief, the 3 surveys adopted stratified three-staged probability sampling scheme. According to the geographic areas and the specific ethnic groups, Taiwan was divided into several strata in the two earlier surveys. In the latest survey, the 20 strata were corresponding to the 20 counties or metropolitan cities. And then, three-stage sampling was carried out in each stratum. First stage was for the selection of primary sampling units (PSU: townships or city districts) via the method of probability proportional to size. Next stage was to randomly choose two initial households within each selected PSU. And the final stage was to do door to door survey in two clusters, starting from each of the two initial households. The protocol and informed consent form have been approved by the Institutional Review Boards (IRB) of Academia Sinica and National Health Research Institutes. A signed informed consent has been obtained from every participant prior to the data collection.

The sample size was 3,071, 1,673 and 1,440 for those who aged equal to or above 19 years old, respectively from the 3 surveys for the prevalence estimation. There were 16 MO (BMI ≥35 kg/m^2^) cases found in NAHSIT 2005–2008 and 23 MO cases in 2013–2014. For epidemiological characteristics comparison, a total of 64 age (±3 years) and gender-matched normal BMI (18.5–24 kg/m^2^) controls were selected in a 4 to 1 ratio [15] for the former survey and 92 controls selected the same way for the latter survey.

### Data collection and derivation of variables of interest

In a face-to-face interview, data on socio-demographics (education, personal income, and occupation), physical activity, and lifestyles (drinking, smoking, betel nut chewing and dietary intake assessed by 24-hour recall and food frequency) were collected. Anthropometric and biochemical profiles were assessed through physical examination.

To appraise energy expenditure from physical activity, we obtained metabolic equivalents of task (METs) for each physical activity and weighted these METs with their corresponding physical activity time [16, 17]. The median MET-minutes per week were compared between NW and MO. Further, we assessed and compared the percentage below the recommendation of physical activity (450 MET-minutes per week) between NW and MO [16, 17].For characterizing smoking, alcohol drinking and betel nut chewing habits, we have three categories for each: namely “never-users” for those who never established the habit, “ex-users” for those who had the habit but had quitted, and “current user” for those who continued with the habit.

Twenty four-hour dietary recall [13]was used to appraise mean dietary nutrient intake levels. We assessed and compared median nutrient density [18](nutrient level divided by the total kcal of an individual and multiplied by 2000) between MO cases and NW controls.

We used information on food frequency to search for dietary pattern associated with obesity. From a total of 72 original food items, we combined them into 21 food categories considering the food groups and nutrient density. And then reduced rank regression (RRR) [19] was applied to generate a linear combination of correlated food frequencies which maximized the BMI variation explained. The factor loading value generated from RRR for each food category (Table C in S1 File) was used to calculate factor loading value-weighted sum of the consumption frequencies, i.e. the dietary pattern score. Since the higher the absolute factor loading values, the greater the association with BMI; food categories with higher absolute factor loading values were considered as influential and used to describe the dietary pattern.

### Statistical analysis

The data was analyzed using SAS statistical software version 9.4 (SAS Inc., NC, USA). All samples in each survey were weighted by individual sample weights to estimate the prevalence rates of underweight, normal weight, overweight, and 3 classes of obesity.

For describing characteristics of the MO and matched NW, frequencies and proportions are presented for categorical variables; so are mean (standard deviation, SD), median, or inter-quartile range (25th to 75th percentiles) for continuous variables. The Chi-square test (for proportion), and/or Mann-Whitney U test (for continuous variables with non-normal distributions) were used when appropriate to test the differences between cases and controls.

The RRR analysis was used to find dietary pattern associated with BMI and to calculate the dietary pattern score as described above. We included in those food categories with absolute factor loading values higher than 0.15 to show the meaning of dietary pattern. And then Spearman correlation was used to examine the correlation between the food frequency and the dietary pattern score derived. Finally, logistic regression was used to assess the associations between morbid obesity and various lifestyle and socioeconomic variables significantly different between MO and NW, including: dietary pattern score, physical inactivity, betel nut chewing, education and personal income.

## Results

### Trends for prevalence rates of overweight, obesity and MO

**Fig 1:**
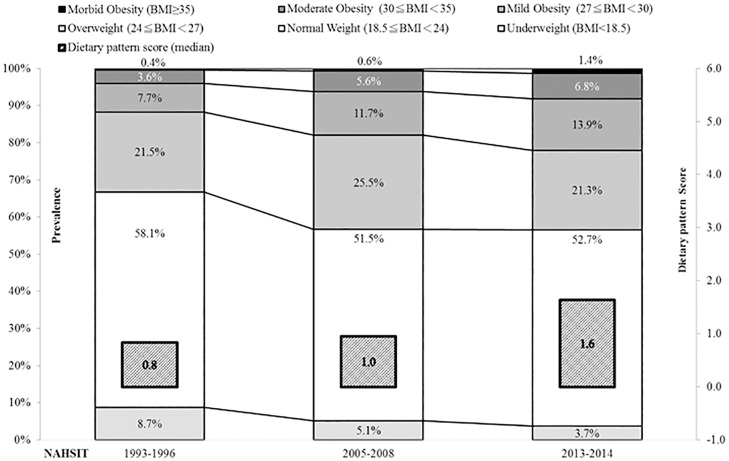
The BMI distribution (proportions of underweight, normal weight, overweight, and several obesity classes) and median dietary pattern score by surveys.

According to 3 waves of the NAHSIT data from 1993–1996, 2005–2008 to the latest 2013–2014 (Fig 1), the percentage of overweight increased from 1993–1996 to 2005–2008, but stabilized from 2005–2008 to 2013. However, both the prevalence rates of obesity (11.8%, 17.9%, to 22.1%) and MO (0.4%, 0.6%, to1.4%) increased sharply in Taiwan.

### Socio-demographic and lifestyle characteristics of the MO

We found that MO status was significantly and negatively associated with the levels of education (p = 0.0247) and income (p = 0.0372) and physical inactivity (< 450 MET-minutes per week) (p = 0.0028), and the betel nut chewing habit (p = 0.0138) (Table 1). However, no significant differences were found between MO cases and NW controls with respect to occupation type, alcohol consumption, smoking status, and sleeping duration; although some unfavorable trends for sleeping time and smoking can be seen for MO.

**Table 1:** Socio-demographic, lifestyle characteristics of the normal weight and the morbid obesity groups.

	Normal Weight ^b^ (18.5≦BMI<24)		Morbid Obesity ^b^ (BMI≧35)		*P*^c^
	n = 156		n = 39		
BMI ^a^ (kg/m^2^)	21.5	1.6	39	2.9	
Age (Years)	36	13.2	35.9	13.4	
19–30 years (%)	51.3		51.3		
30–50 years (%)	25.6		25.6		
50–65 years (%)	23.1		23.1		
Gender					
Female (%)	66.7		66.7		
Male (%)	33.3		33.3		
Education					
Elementary school (%)	9.6		20.5		0.0247^‡^
High schools (%)	43		53.9		
University or above (%)	47.4		25.6		
Occupation					
Blue Collar (%)	55.2		72.7		0.1376^‡^
Low level manager (%)	16.9		6.1		
High level manager (%)	27.9		21.2		
Socioeconomic Level					
Low (%)	36		57.6		0.0760^‡^
Middle (%)	38.2		24.2		
High (%)	25.7		18.2		
Personal Income per month					
Under 30,000 NTD (%)	52.6		73.7		0.0188^‡^
Above 30,000 NTD (%)	47.4		26.3		
Marriage Status					
Single (%)	43.6		41		0.7135^‡^
Married (%)	48.1		53.9		
Divorced/Widow(er)s (%)	8.3		5.1		
Physical Activity (MET-Minutes)	900	(300–2157.5)	397.5	(0.0–1350.0)	0.0084^†^
Under 450 MET-Minutes Per Weeks (%)	30.8		56.4		0.0028^‡^
Sleeping duration (hours)	7.7	(7.0–8.3)	7.7	(6.4–8.5)	0.9060^†^
Under 7 hours (%)	25		35.9		0.1712^‡^
Above 7 hours (%)	75		64.1		
Smoking					
Never (%)	58		40.9		0.3281^‡^
Ex-smoker (%)	13.6		22.7		
Current (%)	28.4		36.4		
Alcohol Drinking					
Never (%)	47.2		46.2		0.9583^‡^
Ex-drinker (%)	2.8		3.9		
Current (%)	50		50		
Betel Nut Chewing					
Never (%)	94.2		81.1		0.0138^‡^
Ex-chewer (%)	3.2		5.4		
Current (%)	2.6		13.5		

^a^ BMI: body mass index.

^b^ Mean (SD) for age and BMI; Median and interquartile range (25th -75th percentile) or %.

^c^ The case and control group were compared with either Mann-Whitney U test(†) or Chi-square test(‡).

### Comparing dietary nutrient intake levels between MO and NW

The median caloric intake per day was higher in MO cases than in NW controls (Table 2). However, the difference was not statistically significant (p = 0.4352). In terms of quality of the diet, fat density was significantly higher; but carbohydrate, dietary fiber and calcium densities were significantly lower in MO cases than in NW controls. Nevertheless, no significant difference was found for nutrient densities of various water- or fat-soluble vitamins.

**Table 2:** Median total caloric intake and nutrient intake levels per 2000 calories by weight status (normal weight vs morbid obesity).

Characteristics ^a^	Normal Weight ^b^ (18.5≤BMI<24)		Morbid Obesity ^b^ (BMI≥35)		*P*^c^
	n = 156		n = 39		
Calorie (kcal/day)	1917.6	(1397.2–2511.0)	2111.5	(1489.5–2617.2)	0.4352
Carbohydrate (g)	263.3	(226.8–297.9)	236.3	(195.3–272.8)	0.0091
Protein (g)	81.0	(67.8–95.3)	84.1	(62.9–94.4)	0.7887
Fat (g)	69.0	(56.1–81.3)	80.1	(66.8–97.8)	0.0009
Cholesterol (mg)	297.9	(147.7–466.5)	385.0	(135.1–479.2)	0.2974
Dietary fiber (g)	16.6	(10.8–23.7)	12.8	(7.9–21.7)	0.0337
Vit. B1 (mg)	1.1	(0.8–1.6)	1.2	(0.9–1.6)	0.2166
Vit. B2 (mg)	1.2	(0.9–1.6)	1.1	(0.8–1.5)	0.4342
Nicotinic acid (mg)	18.3	(13.2–23.6)	19.2	(15.3–24.3)	0.3839
Vit. B6 (mg)	1.7	(1.3–2.4)	1.8	(1.3–2.2)	0.9078
Vit. B12 (μg)	3.1	(1.9–6.2)	3.1	(2.1–6.2)	0.7236
Vit. C (mg)	125.2	(59.5–223.6)	125.7	(40.5–252.0)	0.9507
Vit. A (ug)	579.1	(333.7–1039.0)	629.8	(306.5–1243.9)	0.9456
Vit. D (μg)	3.6	(1.8–7.5)	3.2	(1.3–9.1)	0.3815
Vit. E (mg)	8.2	(5.9–10.7)	8.5	(5.8–10.6)	0.6650
Na (g)	3.6	(2.7–4.5)	4.0	(2.8–5.7)	0.1854
K (g)	2.5	(1.9–3.4)	2.4	(1.9–3.1)	0.3961
Mg (mg)	281.4	(205.8–350.8)	254.1	(195.2–324.1)	0.3278
Ca (mg)	471.7	(311.3–749.0)	410.1	(203.1–548.9)	0.0271
P (mg)	1131.3	(958.7–1455.8)	1119.7	(914.7–1247.1)	0.1060
Zn (mg)	10.4	(8.3–13.1)	10.1	(8.4–13.3)	0.8280
Fe (mg)	14.2	(10.7–18.3)	13.3	(9.8–17.0)	0.3093

^a^ BMI: body mass index; Vit.: vitamin; Na: sodium; K: potassium; Mg: magnesium; Ca: calcium; P: phosphorus; Zn: zinc; Fe: iron.

^b^ Median and interquartile range (25th - 75th percentile).

^c^ The case and control group were compared with Mann-Whitney U test.

### Dietary pattern associated with MO

The frequencies of these food categories were all significantly (p<0.05) correlated with the dietary pattern score (Table 3). Therefore, this BMI associated dietary pattern was featured with lower frequencies of fresh fruits and 100% juice; nuts; milk, yogurt, and cheese; breakfast cereals; and beverage without sugar (coffee and tea); but higher frequencies of red meat; processed seafood and meat products; ice pop, candy, and sweetened beverage.

**Table 3:** Food items with high absolute loading values discovered by RRR analysis, correlation coefficients between food frequency and dietary pattern score, and median food frequency by weight status.

Food item	RRR		Median food intake frequency per month		
	Loading value ^a^	Correlation coefficient ^b^	Normal Weight (18.5≤BMI<24) n = 156	Morbid Obesity (BMI≥35) n = 39	P ^c^
Ice pop, candy, sweetened beverage	0.42	0.68***	5.0	8.2	0.1562
Red meat	0.24	0.30***	3.0	5.0	0.0726
Processed seafood & meat products	0.17	0.28**	1.2	1.2	0.4697
Tea & Coffee (no sugar)	-0.16	-0.38***	1.0	0.0	0.2049
Breakfast cereals	-0.20	-0.28**	0.0	0.0	0.2237
Milk, Yogurt, Cheese	-0.28	-0.41***	1.4	0.8	0.1124
Nuts	-0.35	-0.25**	0.3	0.0	0.0209
Fresh fruit & 100% juice	-0.36	-0.52***	5.2	3.7	0.0345

^a^ The factor loading was calculated by reduced rank regression (RRR).

^b^ The correlation coefficient between food frequency and dietary pattern score was calculated by Spearman correlation.

*P-value <0.05;

**P-value <0.001;

***P-value <0.0001

^c^ The case and control group were compared by using Mann-Whitney U test.

For comparing frequency of single food item between MO and NW (Table 3), fresh fruits and 100% juice (p = 0.0345) and nuts (p = 0.0209) were consumed significantly more by NW controls than MO cases. In contrast, red meat (p = 0.0726) were consumed more by MO cases than NW controls at a borderline significance level. Other items also showed the trends in line with the dietary pattern, but significance was not achieved.

Finally, we examined the independent effect of dietary pattern score on MO status (Table 4). In univariate analysis, we found that dietary pattern score, physical inactivity, and low levels of education and income were associated with MO, respectively with age and sex controlled. Betel nut chewing was not (data not shown). However, when both dietary pattern factor and physical inactivity were included in the model (model 1), only dietary pattern score was significant. If we further included in education and income variables (model 2), the significance remained for the dietary pattern score with a significant trend (p for trend = 0.0002). The median of dietary pattern score increased steadily from 1993–1996, to 2005–208, and to 2013–2014 (see Fig 1).

**Table 4:** The Odds ratio of quartiles of dietary pattern score to morbid obesity.

	Univariate		Model 1.		Model 2.	
	OR	95% CI	OR	95% CI	OR	95% CI
Dietary pattern scores^a^						
Q1	1.0		1.0		1.0	
Q2	0.9	(0.2–4.6)	0.9	(0.2–4.7)	0.8	(0.1–4.0)
Q3	5.4	(1.5–20.1)	4.7	(1.2–17.9)	3.9	(1.0–15.6)
Q4	6.7	(1.8–24.8)	5.8	(1.5–22.2)	4.7	(1.2–19.0)
Physical Activity						
MET-Minutes per week<450	2.8	(1.3–6.0)	2.1	(1.0–4.8)	1.9	(0.8–4.5)
450≦MET-Minutes per week	1.0		1.0		1.0	
Education level						
Elementary school	6.9	(1.8–26.2)			4.0	(0.9–17.6)
High schools	2.1	(0.9–5.0)			1.7	(0.7–4.3)
University or above	1.0				1.0	
Personal income						
Under 30,000 NTD	3.0	(1.2–7.2)			2.2	(0.8–5.9)
Above 30,000 NTD	1.0				1.0	
p for trend of dietary pattern scores	0.0008		0.0025		0.0071	

Total N = 175. 16 subjects (12 controls and 4 cases) were excluded due to missing values on FFQ.

^a^ The dietary pattern score was the weighted sum of food frequencies, weighted by loading factors generated from RRR.

Univariate: age and sex-controlled. Model 1: dietary pattern scores and physical activity controlled for age and sex. Model 2: Model 1 adjusted further for education level, and personal income.

## Discussion

Over past four decades, global age-standardized prevalence of obesity (BMI≥30 kg/m^2^) increased from 3.2% to 10.8% in men, and from 6.4% to 14.9% in women [20]. And in Taiwan, the prevalence trend of overweight or obesity (BMI≥24 kg/m^2^) together has increased from 33.2%to 43.4% (1993–1996 to 2005–2008) and remained that level in latest survey. However, the prevalence trend of obesity (BMI≥27 kg/m^2^) was continuously increasing from 11.8%, 17.9%, and reached 22.0% in 2013–2014. Moreover, there was a noticeable increase in the MO (BMI≥35 kg/m^2^) prevalence in the same period, from 0.4% to more than one and half percentage. However, prevalence of overweight decreased from 25.5% during the 2005–2008 survey to the recent 21.3%. These findings suggest that, although the prevalence stabilized for overweight and obesity as a whole, the prevalence of obesity including the “morbid obesity" is dramatically rising. Similar observations were also seen recently in European countries, US, Sweden, and China [4, 12, 21–23]. It is likely that this phenomenon of BMI polarization in Taiwan is due in part to the shifting to the desirable weight range for those who were at the lower end of the overweight range, since the shrinkage of overweight proportion occurred clearly after the nationwide campaign on "Losing 6 Million Tons" in 2011[24] and mostly health conscious people participated in the movement. What happened at the other end of the spectrum seems to show that the obesogenic environment continues to pull more toward BMI beyond the obesity and the MO cut-points. We have demonstrated that MO cases have much worse clinical chemistry and metabolic profiles than their non-obese counter parts(Table B in S1 File), similar to many other reports[25]. One should not overlook the threat of MO for health and economics in the years to come [26] and it is crucial to understand the epidemiological characteristics of the MO in order to make effective strategic plans.

We have found that MO subjects tended to be the under-privileged, i.e., with lower level of education and income. Their low physical activity and poor dietary pattern are in part associated with their socioeconomic status (SES). The relationship between SES and obesity has been widely studied [27–29]. In the developing countries, obesity tends to be positively associated with SES. However, in the developed countries, the directions of the association are mixed. In addition, across different socioeconomic categories, the obesity prevalence seems to rise with time in the group with lower education level, and remains stable or increases slightly in the group with higher education level[30, 31]. The association between MO and low SES may indicate that Taiwanese have experienced the nutrition transition. As economy developed, the dietary pattern also changed from whole foods and plant foods-rich one into those abundant with animal products and processed foods high in fats and sugar[32, 33]. Therefore, it would lead to obesity and diet-related non-communicable diseases[34, 35]. This transition has been observed in many countries in Asia too [36].

It is crucial to understand the nutrient vectors or food vectors contributing to obesity or MO for developing effective health policies. Dietary pattern analysis for obesity or for incremental BMI gained much attention recently [37]. Most of the dietary pattern studies [38–40] employed principle component (or factor) analysis and obtained healthy (prudent) vs. western (animal protein) patterns or traditional vs. modern patterns. Western, modern, and animal protein pattern concurred with higher risk, but healthy and traditional ones with lower risk of obesity. Only a few studies in Greece[41] and in Australia [42] applied RRR method in recent years, identifying a pattern featured with increased sweets/SSB and red/processed meat, but lowered fruits and vegetables. Our study is the first studying Asians. Our findings on nutrient density from 24-hour recall and on food pattern analysis with food frequency questionnaire are consistent between each other and with the previous mentioned RRR studies. According to the 24-hour recall, the MO subjects tend to consume a diet high in fat; but low in calcium, fiber and carbohydrate. From dimension reduction analysis of the food frequency data, the diet of Taiwanese MO was composed of less fresh fruit, nuts, breakfast cereals, and dairy products; but of more red meat, processed animal products, and sweets/sweetened beverage. These findings on diet are consistent with the obesogenic dietary pattern discovered previously, i.e., abundant with animal products and processed foods high in fat and simple sugar [32, 34–43], although percent energy from carbohydrate is relatively less in the MO subjects of our study. It is interesting to observe that some not commonly consumed food items such as nuts, breakfast cereal and dairies are beneficial in Taiwan. High consumption frequency of sugar-sweetened beverages (SSBs) has been associated with some adverse health consequences, i.e., obesity, metabolic syndromes, gout, and non-alcoholic fatty liver disease [44]. Our data showed that it is the dietary pattern with less nutrient dense foods and more SSBs which made the contributions. Although it is probably hard to tease apart the contributions from lack of nutrient-dense foods, our findings suggest that the key problem may be accessibility and affordability of the healthy food (wholegrain cereals, fruits and vegetables) rather than availability of foods in Taiwan nowadays[45, 46]. We were able to show that median of BMI-associated dietary pattern score increased steadily from 1990’s to 2010’s (0.8 →1.0 →1.6), indicating a shift toward obesogenic dietary pattern. Effective public health measures are required to educate people and to build healthy eating environment.

We also found that low physical activity was significantly associated with MO. Although, it was hard to separate the effect of diet and physical activity, there are large declines in level of physical activity and increases in sedentary behavior globally[47]. Lack of sufficient physical activity has been viewed as a major crisis of public health[48, 49], including data from Taiwan [50] and China [51]. Unfortunately, we could not examine the trend of physical inactivity due to different questionnaires employed in three surveys.

This is the first study portraying MO epidemiology in an Asian population, providing not only the descriptive statistics but also socio-demographic and lifestyle determinants of the MO. However, there are some limitations in this study. First of all, the sample size of MO and the power of the study are relatively small. We did not observe an effect from sleeping duration, and caloric intake due to low statistical power contributed by small sample size, relatively small effect and large variation of the variables. Nevertheless, our study provides a first glance on epidemiological characteristics of MO in an Asian population. Secondly, some of the discovered associations between MO and epidemiological characteristics are cross-sectional in nature. Cautions should be taken to interpret the findings, in particularly on the negative association between physical activity and MO status. Although physical inactivity may contribute to positive energy balance leading to obesity, MO subjects may also have difficulty to carry out physical activity. Furthermore, genetic factors may contribute to the development of obesity and morbid obesity. Individual with the genetic susceptibility may be more so influenced[52] by obesogenic environment[53] such as the accessibility of processed and high-energy-dense foods, and desk-constrained works as a consequence of modernization and social development. The risk factors of MO found in this study were exactly what resulted from the obesogenic environments. However, it is lacking genetic epidemiology study on MO, especially in Asian populations. Further research on genetics of MO in Asian populations is needed. We could not investigate the genetic and environmental interactions in this study, since genetic component was not included in the IRB approval.

In summary, this study illustrates how MO and desirable weight polarized recently in Taiwan. In addition, comprehensive epidemiological characteristics of MO have been studied. MO primarily appears among those who had lower education and personal income. They were physically inactive and tend to consume a nutrient-poor dietary pattern which are high in red meat/processed animal products and sweets/sweeten beverage, but less in fruits, nuts, and vegetables. Our results point to the role of poor lifestyle and associated obesogenic environmental factors in contributing to the development of MO in those of the underprivileged, which have important implications in developing national health policy.

## Supporting information

S1 File Definition and Database. (PDF)

## References

[1] (WHO) WHO. Facts Sheet N°311, Obesity and overweight.: World Health Organization (WHO); 2015 [cited 2016 March]. http://www.who.int/mediacentre/factsheets/fs311/en/.

[2] Haslam DW, James WP. Obesity. Lancet. 2005;366(9492):1197–209. Epub 2005/10/04. 16198769 10.1016/S0140-6736(05)67483-1

[3] Visscher TL, Seidell JC. The public health impact of obesity. Annu Rev Public Health. 2001;22:355–75. Epub 2001/03/29. 11274526 10.1146/annurev.publhealth.22.1.355

[4] Rokholm B, Baker JL, Sorensen TI. The levelling off of the obesity epidemic since the year 1999—a review of evidence and perspectives. Obesity reviews: an official journal of the International Association for the Study of Obesity. 2010;11(12):835–46. Epub 2010/10/27. 20973911 10.1111/j.1467-789X.2010.00810.x

[5] Finkelstein EA, Khavjou OA, Thompson H, Trogdon JG, Pan L, Sherry B, et al Obesity and severe obesity forecasts through 2030. Am J Prev Med. 2012;42(6):563–70. Epub 2012/05/23. 10.1016/j.amepre.2011.10.026 22608371

[6] Twells LK, Gregory DM, Reddigan J, Midodzi WK. Current and predicted prevalence of obesity in Canada: a trend analysis. CMAJ Open. 2014;2(1):E18–26. 10.9778/cmajo.20130016 25077121 PMC3985909

[7] Bray GA. Medical consequences of obesity. J Clin Endocrinol Metab. 2004;89(6):2583–9. Epub 2004/06/08. 15181027 10.1210/jc.2004-0535

[8] Prospective Studies C, Whitlock G, Lewington S, Sherliker P, Clarke R, Emberson J, et al Body-mass index and cause-specific mortality in 900 000 adults: collaborative analyses of 57 prospective studies. Lancet. 2009;373(9669):1083–96. Epub 2009/03/21. 10.1016/S0140-6736(09)60318-4 19299006 PMC2662372

[9] Pan WH, Yeh WT, Chen HJ, Chuang SY, Chang HY, Chen L, et al The U-shaped relationship between BMI and all-cause mortality contrasts with a progressive increase in medical expenditure: a prospective cohort study. Asia Pac J Clin Nutr. 2012;21(4):577–87. 23017316

[10] Withrow D, Alter DA. The economic burden of obesity worldwide: a systematic review of the direct costs of obesity. Obesity reviews: an official journal of the International Association for the Study of Obesity. 2011;12(2):131–41. 20122135 10.1111/j.1467-789X.2009.00712.x

[11] Colquitt JL, Pickett K, Loveman E, Frampton GK. Surgery for weight loss in adults. Cochrane Database Syst Rev. 2014;8:CD003641. Epub 2014/08/12. 10.1002/14651858.CD003641.pub4 PMC9028049 25105982

[12] Sturm R. Increases in morbid obesity in the USA: 2000–2005. Public health. 2007;121(7):492–6. 10.1016/j.puhe.2007.01.006 17399752 PMC2864630

[13] Tu SH, Chen C, Hsieh YT, Chang HY, Yeh CJ, Lin YC, et al Design and sample characteristics of the 2005–2008 Nutrition and Health Survey in Taiwan. Asia Pac J Clin Nutr. 2011;20(2):225–37. Epub 2011/06/15. 21669592

[14] Pan W-H, Kao M-D, Tzeng M-S, Yen L-L, Hung Y-T, Li L-A, et al Nutrition and Health Survey in Taiwan (NAHSIT) 1993–1996: Design, Contents, and Operations. Nutr Sci J. 1999;24(1):1–10.

[15] Hennekens CH B J, Mayrent SL. Epidemiology in medicine. Boston: Little, Brown & Co.; 1987.

[16] Sallis JF, Haskell WL, Wood PD, Fortmann SP, Rogers T, Blair SN, et al Physical activity assessment methodology in the Five-City Project. Am J Epidemiol. 1985;121(1):91–106. 3964995 10.1093/oxfordjournals.aje.a113987

[17] Haskell WL, Lee IM, Pate RR, Powell KE, Blair SN, Franklin BA, et al Physical activity and public health: updated recommendation for adults from the American College of Sports Medicine and the American Heart Association. Circulation. 2007;116(9):1081–93. Epub 2007/08/03. 17671237 10.1161/CIRCULATIONAHA.107.185649

[18] Willett W, Stampfer MJ. Total energy intake: implications for epidemiologic analyses. Am J Epidemiol. 1986;124(1):17–27. Epub 1986/07/01. 3521261 10.1093/oxfordjournals.aje.a114366

[19] Hoffmann K, Schulze MB, Schienkiewitz A, Nothlings U, Boeing H. Application of a new statistical method to derive dietary patterns in nutritional epidemiology. Am J Epidemiol. 2004;159(10):935–44. 15128605 10.1093/aje/kwh134

[20] NCD-RisC. Trends in adult body-mass index in 200 countries from 1975 to 2014: a pooled analysis of 1698 population-based measurement studies with 19.2 million participants. Lancet. 2016;387(10026):1377–96. Epub 2016/04/27. 10.1016/S0140-6736(16)30054-X 27115820 PMC7615134

[21] Lao X, Ma W, Sobko T, Zhang Y, Xu Y, Xu X, et al Overall obesity is leveling off while abdominal obesity continues to rise in a chinese population experiencing rapid economic development: Analysis of serial cross-sectional health survey data 2002–2010. International Journal of Obesity. 2014. 10.1038/ijo.2014.95 24858655

[22] Lien N, Henriksen HB, Nymoen LL, Wind M, Klepp KI. Availability of data assessing the prevalence and trends of overweight and obesity among European adolescents. Public Health Nutr. 2010;13(10a):1680–7. 10.1017/S1368980010002223 20883566

[23] Neovius M, Teixeira-Pinto A, Rasmussen F. Shift in the composition of obesity in young adult men in Sweden over a third of a century. Int J Obes (Lond). 2008;32(5):832–6. 18087264 10.1038/sj.ijo.0803784

[24] Tucker C. Taiwan weight-loss program helps country shed 2,000 tons. The Nation's Health. 2013;43(4):23.

[25] Martin-Rodriguez E, Guillen-Grima F, Marti A, Brugos-Larumbe A. Comorbidity associated with obesity in a large population: The APNA study. Obes Res Clin Pract. 2015;9(5):435–47. 10.1016/j.orcp.2015.04.003 25979684

[26] Visscher TL, Heitmann BL, Rissanen A, Lahti-Koski M, Lissner L. A break in the obesity epidemic? Explained by biases or misinterpretation of the data? Int J Obes (Lond). 2015;39(2):189–98. Epub 2014/06/10. 24909829 10.1038/ijo.2014.98

[27] Wang Y, Beydoun MA. The obesity epidemic in the United States—gender, age, socioeconomic, racial/ethnic, and geographic characteristics: a systematic review and meta-regression analysis. Epidemiologic reviews. 2007;29(1):6–28. 17510091 10.1093/epirev/mxm007

[28] Hou X, Jia W, Bao Y, Lu H, Jiang S, Zuo Y, et al Risk factors for overweight and obesity, and changes in body mass index of Chinese adults in Shanghai. BMC Public Health. 2008;8(1):389. 19025585 10.1186/1471-2458-8-389 PMC2632663

[29] Dinsa GD, Goryakin Y, Fumagalli E, Suhrcke M. Obesity and socioeconomic status in developing countries: a systematic review. Obesity reviews: an official journal of the International Association for the Study of Obesity. 2012;13(11):1067–79. 22764734 10.1111/j.1467-789X.2012.01017.x PMC3798095

[30] Marques-Vidal P, Bovet P, Paccaud F, Chiolero A. Changes of overweight and obesity in the adult Swiss population according to educational level, from 1992 to 2007. BMC Public Health. 2010;10(1):87. 20170554 10.1186/1471-2458-10-87 PMC2831837

[31] Lahti‐Koski M, Harald K, Saarni S, Peltonen M, Männistö S. Changes in body mass index and measures of abdominal obesity in Finnish adults between 1992 and 2007, the National FINRISK Study. Clinical Obesity. 2012;2(1‐2):57–63. 10.1111/j.1758-8111.2012.00035.x 25586048

[32] Hawkes C. Uneven dietary development: linking the policies and processes of globalization with the nutrition transition, obesity and diet-related chronic diseases. Global Health. 2006;2(1):4. 16569239 10.1186/1744-8603-2-4 PMC1440852

[33] Popkin BM, Adair LS, Ng SW. Global nutrition transition and the pandemic of obesity in developing countries. Nutr Rev. 2012;70(1):3–21. 10.1111/j.1753-4887.2011.00456.x 22221213 PMC3257829

[34] (WHO) WHO. Diet, nutrition and the prevention of chronic diseases: report of a Joint WHO/FAO Expert Consultation. Geneva, Switzerland: World Health Organization (WHO) 2002 31/12/2015.

[35] S Ba, S JC, J WPT. Diet, nutrition and the prevention of excess weight gain and obesity. Public Health Nutr. 2004;7(1a):123–46. 14972057 10.1079/phn2003585

[36] Pingali P. Westernization of Asian diets and the transformation of food systems: Implications for research and policy. Food Policy. 2007;32(3):281–98.

[37] Fung TT, Rimm EB, Spiegelman D, Rifai N, Tofler GH, Willett WC, et al Association between dietary patterns and plasma biomarkers of obesity and cardiovascular disease risk. Am J Clin Nutr. 2001;73(1):61–7. Epub 2000/12/22. 11124751 10.1093/ajcn/73.1.61

[38] Bell LK, Edwards S, Grieger JA. The Relationship between Dietary Patterns and Metabolic Health in a Representative Sample of Adult Australians. Nutrients. 2015;7(8):6491–505. Epub 2015/08/08. 10.3390/nu7085295 26251918 PMC4555134

[39] San-Cristobal R, Navas-Carretero S, Celis-Morales C, Brennan L, Walsh M, Lovegrove JA, et al Analysis of Dietary Pattern Impact on Weight Status for Personalised Nutrition through On-Line Advice: The Food4Me Spanish Cohort. Nutrients. 2015;7(11):9523–37. Epub 2015/11/26. 10.3390/nu7115482 26593942 PMC4663610

[40] Shu L, Zheng PF, Zhang XY, Si CJ, Yu XL, Gao W, et al Association between Dietary Patterns and the Indicators of Obesity among Chinese: A Cross-Sectional Study. Nutrients. 2015;7(9):7995–8009. Epub 2015/09/24. 10.3390/nu7095376 26393646 PMC4586571

[41] Manios Y, Kourlaba G, Grammatikaki E, Androutsos O, Ioannou E, Roma-Giannikou E. Comparison of two methods for identifying dietary patterns associated with obesity in preschool children: the GENESIS study. Eur J Clin Nutr. 2010;64(12):1407–14. Epub 2010/09/03. 10.1038/ejcn.2010.168 20808335

[42] Appannah G, Pot GK, Huang RC, Oddy WH, Beilin LJ, Mori TA, et al Identification of a dietary pattern associated with greater cardiometabolic risk in adolescence. Nutr Metab Cardiovasc Dis. 2015;25(7):643–50. Epub 2015/06/01. 10.1016/j.numecd.2015.04.007 26026208 PMC4510146

[43] Popkin B. Synthesis and implications: China's nutrition transition in the context of changes across other low‐and middle‐income countries. Obesity Reviews. 2014;15(S1):60–7. 24341759 10.1111/obr.12120 PMC3869101

[44] Bray GA, Popkin BM. Dietary sugar and body weight: have we reached a crisis in the epidemic of obesity and diabetes?: health be damned! Pour on the sugar. Diabetes care. 2014;37(4):950–6. Epub 2014/03/22. 10.2337/dc13-2085 24652725 PMC9514031

[45] Drewnowski A, Darmon N. The economics of obesity: dietary energy density and energy cost. Am J Clin Nutr. 2005;82(1 Suppl):265S–73S. 16002835 10.1093/ajcn/82.1.265S

[46] Giskes K, Avendano M, Brug J, Kunst AE. A systematic review of studies on socioeconomic inequalities in dietary intakes associated with weight gain and overweight/obesity conducted among European adults. Obesity reviews: an official journal of the International Association for the Study of Obesity. 2010;11(6):413–29. Epub 2009/11/06. 19889178 10.1111/j.1467-789X.2009.00658.x

[47] Ng SW, Popkin BM. Time use and physical activity: a shift away from movement across the globe. Obesity Reviews. 2012;13(8):659–80. 10.1111/j.1467-789X.2011.00982.x 22694051 PMC3401184

[48] Thorp AA, Owen N, Neuhaus M, Dunstan DW. Sedentary behaviors and subsequent health outcomes in adults: a systematic review of longitudinal studies, 1996–2011. American journal of preventive medicine. 2011;41(2):207–15. 10.1016/j.amepre.2011.05.004 21767729

[49] Lim SS, Vos T, Flaxman AD, Danaei G, Shibuya K, Adair-Rohani H, et al A comparative risk assessment of burden of disease and injury attributable to 67 risk factors and risk factor clusters in 21 regions, 1990–2010: a systematic analysis for the Global Burden of Disease Study 2010. Lancet. 2012;380(9859):2224–60. Epub 2012/12/19. 10.1016/S0140-6736(12)61766-8 23245609 PMC4156511

[50] Wen CP, Wai JP, Tsai MK, Yang YC, Cheng TY, Lee MC, et al Minimum amount of physical activity for reduced mortality and extended life expectancy: a prospective cohort study. Lancet. 2011;378(9798):1244–53. 10.1016/S0140-6736(11)60749-6 21846575

[51] Ng SW, Norton EC, Guilkey DK, Popkin BM. Estimation of a dynamic model of weight. Empirical Economics. 2012;42(2):413–43.

[52] Bouchard C. Current understanding of the etiology of obesity: genetic and nongenetic factors. The American journal of clinical nutrition. 1991;53(6):1561S–5S. 2031488 10.1093/ajcn/53.6.1561S

[53] Lee YS. The role of genes in the current obesity epidemic. Ann Acad Med Singapore. 2009;38(1):45–3. Epub 2009/02/18. 19221670

