# Supplementary material for: Morbid obesity in Taiwan: Prevalence, trends, associated social demographics, and lifestyle factors
Source: PLoS One. 2017 Feb 2;12(2):e0169577. doi: 10.1371/journal.pone.0169577 (PMC5289420; doi:10.1371/journal.pone.0169577)
Supplement: S1 File — (PDF) [file pone.0169577.s001.pdf]

Supporting Information

Appendix S1. Definition of adults' metabolic risk factors in Taiwan

Metabolic syndrome was defined when 3 of the following 5 components were fulfilled[1]: central obesity (waist circumference≥90 cm for male and ≥80 cm for female), impaired fasting glucose (FG) (≥100 mg/dL), hypertriglyceridemia (triglyceride, TG ≥150 mg/dL), decreased serum high-density lipoprotein (HDL-C) (<40 mg/dL in males and <50 mg/dL in females), and elevated blood pressure (systolic blood pressure, SBP≥130 mmHg or diastolic blood pressure, DBP≥85 mmHg). Further clinical chemistry definitions include hypercholesterolemia (total cholesterol, TC≥220 mg/dL), elevated low-density lipoprotein cholesterol (LDL-C) (≥130 mg/dL) and hyperuricemia (uric acid, UA≥7 mg/dL for male and ≥6 mg/dL for female).

Table A. Definition of adults' obesity in Taiwan.

Category	BMI (kg/m^2)
Underweight	BMI<18.5
Normal weight	18.5≤BMI<24
Overweight	24≤BMI<27
Obesity (BMI≥27)	Mild obesity: 27≤BMI<30
	Moderate obesity: 30≤BMI<35
	Morbid obesity: BMI≥35

BMI: body mass index.

BMI was defined as weight (kg) divided by height square (m^2). According to the Taiwan Ministry of Health and Welfare[2], the cut-offs value for overweight and obesity are lower than those from WHO.

Table B. Anthropometric and biochemical characteristics of the normal weight and the morbid obesity groups.

Characteristics [a]	Normal Weight [b] (18.5≤BMI<24) n=156		Morbid Obesity [b] (BMI≥35) n=39		P [c]
Waist Circumference (cm)	74.1	(69.5 - 79.5)	112.5	(103.0 - 118.3)	<0.0001†
Central Obesity (%)	9.7		100.0		<0.0001‡
Hip Circumference (cm)	91.6	(88.0 - 95.5)	117.8	(113.2 - 122.8)	<0.0001†
FG (mg/dl)	93.0	(89.0 - 99.0)	105.5	(95.5 - 113.0)	<0.0001†
Impaired FG (%)	24.0		69.4		<0.0001‡
TG (mg/dl)	77.5	(55.0 - 103.5)	124.0	(97.0 - 172.5)	<0.0001†
Hyper TG (%)	9.2		36.1		<0.0001‡
TC (mg/dl)	180.0	(157.0 - 207.0)	185.0	(168.0 - 214.0)	0.1894†
Hypercholesterolemia (%)	14.4		22.2		0.2466‡
HDL-C (mg/dl)	58.0	(50.0 - 68.0)	43.5	(38.0 - 56.0)	<0.0001†
Reduced HDL-C (%)	15.7		52.8		<0.0001‡
LDL-C (mg/dl)	111.0	(90.0 - 135.0)	119.0	(100.5 - 139.0)	0.1051†
Hyper LDL-C (%)	30.7		33.3		0.7607‡
SBP (mmHg)	107.0	(99.0 - 116.0)	128.8	(113.0 - 137.0)	<0.0001†
DBP (mmHg)	68.0	(62.0 - 73.0)	79.0	(71.0 - 87.3)	<0.0001†
Elevated BP (%)	3.9		52.6		<0.0001‡
Metabolic Syndrome (%)	5.1		66.7		<0.0001‡
UA (mg/dl)	5.1	(4.2 - 6.1)	6.6	(5.5 - 7.4)	<0.0001†
Hyperuricemia (%)	19.0		61.1		<0.0001‡

[a] BMI: body mass index; TG: triglyceride; TC: total cholesterol;

HDL-C: high-density lipoprotein; LDL-C: low-density lipoprotein; FG: fasting glucose;

SBP: systolic blood pressure; DBP: diastolic blood pressure; UA: uric acid.

[b] Median and interquartile range (25th -75th percentile) or prevalence (%).

[c] The case and control groups were compared with either Mann-Whitney U test(†) or Chi-square test(‡).

As expected, MO cases had significantly ($p<0.0001$)higher levels of waist and hip circumferences, FG, TG, SBP, DBP and UA and lower level of HDL-C than NW controls. The proportion with metabolic syndrome was also much higher in MO cases than in NW controls (66.7% vs. 5.1%) ($p<0.0001$).

Table C. Factor loading and correlation coefficient of 21 food categories.

21 Food categories	Factor loading [a]	Correlation Coefficient [b]	p [c]
Ice pop, candy, Sweetern beveverage	0.42	0.68	<0.0001
Red Meat	0.24	0.30	<0.0001
Egg	0.19	0.05	0.5117
Processed seafood & meat products	0.17	0.28	0.0002
Pickel Vagetables	0.09	0.11	0.1347
Poultry	0.08	0.17	0.0202
Roe and innards	0.06	0.11	0.1523
Rice milk	0.03	0.05	0.5060
Soybean products	0.00	-0.04	0.5881
Flavored creamy dairy products	-0.06	0.01	0.9115
Vegetables	-0.07	-0.26	0.0003
Seafood	-0.09	-0.15	0.0384
Herbal tea	-0.13	0.00	0.9609
Tea Coffee (no sugar)	-0.16	-0.38	<0.0001
Breakfast cereals	-0.20	-0.28	0.0001
Sweetened starchy bean products	-0.22	-0.04	0.6050
Milks, Ypgurts, Cheese	-0.28	-0.41	<0.0001
Rice & Noodle Products	-0.32	-0.13	0.0926
Roots & Tubers	-0.34	-0.15	0.0512
Nuts	-0.35	-0.25	0.0007
Fresh fruit & 100% juice	-0.36	-0.52	<0.0001

[a] The factor loading was calculated by reduced rank regression (RRR).

[b] The correlation coefficient between food frequency and dietary pattern score was calculated by Spearman correlation.

[c] The case and control group were compared by using Mann-Whitney U test.

In this study, we have grouped 72 original food items into 21 food categories by food groups and nutrient density. In this table we show the detailed results of 21 food categories form RRR. And the correlation between intake frequency per month of 21 food categories and dietary pattern score.

Reference

1. Health Promotion Administration (HPA) MoHaWM. Definition of adults metabolic syndrome in Taiwan.: Health Promotion Administration (HPA), Ministry of Health and Welfare (MOHFW); 2007 [updated 30/01; cited 2016 March]. Available from: http://www.hpa.gov.tw/BHPNet/Web/HealthTopic/TopicArticle.aspx?No=200712250123&parentid=200712250023.

2. (DOH) DoH. Definition of adults obesity in Taiwan Department of Health, Executive Yuan: Department of Health (DOH), Executive Yuan; 2007 [cited 2016 March]. Available from: http://health99.hpa.gov.tw/OnlinkHealth/BMI.html.
